# A Conceptual Framework to Integrate Biodiversity, Ecosystem Function, and Ecosystem Service Models

**DOI:** 10.1093/biosci/biac074

**Published:** 2022-09-01

**Authors:** Sarah R Weiskopf, Bonnie J E Myers, Maria Isabel Arce-Plata, Julia L Blanchard, Simon Ferrier, Elizabeth A Fulton, Mike Harfoot, Forest Isbell, Justin A Johnson, Akira S Mori, Ensheng Weng, Zuzana V HarmáCˇková, María Cecilia Londoño-Murcia, Brian W Miller, Laura M Pereira, Isabel M D Rosa

**Affiliations:** US Geological Survey National Climate Adaptation Science Center, in Reston, Virginia, United States; North Carolina State University, Raleigh, North Carolina, United States; Instituto de Investigación de Recursos Biológicos Alexander von Humboldt, Bogotá, Colombia; University of Tasmania, Hobart, Tasmania, Australia; Land and Water, CSIRO, Canberra, Australian Capital Territory, Australia; University of Tasmania, Hobart, Tasmania, Australia; United Nations Environment Programme–World Conservation Monitoring Centre, Cambridge, England, United Kingdom; University of Minnesota, Saint Paul, Minnesota, United States; University of Minnesota, Saint Paul, Minnesota, United States; University of Tokyo, Meguro, Tokyo, Japan; Columbia University and with the NASA Goddard Institute for Space Studies, both New York, New York, United States; Czech Academy of Sciences, Brno, Czechia and with the Stockholm Resilience Centre, Stockholm University, Stockholm, Sweden; Instituto de Investigación de Recursos Biológicos Alexander von Humboldt, Bogotá, Colombia; US Geological Survey North Central Climate Adaptation Science Center, Boulder, Colorado, United States; University of the Witwatersrand, Johannesburg, South Africa and with the Stockholm Resilience Centre, Stockholm University, Stockholm, Sweden; Bangor University, Gwynedd, Wales, United Kingdom

**Keywords:** biodiversity, biodiversity–ecosystem function relationships, ecosystem function, ecosystem services, modeling, sustainability, trait-based modeling

## Abstract

Global biodiversity and ecosystem service models typically operate independently. Ecosystem service projections may therefore be overly optimistic because they do not always account for the role of biodiversity in maintaining ecological functions. We review models used in recent global model intercomparison projects and develop a novel model integration framework to more fully account for the role of biodiversity in ecosystem function, a key gap for linking biodiversity changes to ecosystem services. We propose two integration pathways. The first uses empirical data on biodiversity–ecosystem function relationships to bridge biodiversity and ecosystem function models and could currently be implemented globally for systems and taxa with sufficient data. We also propose a trait-based approach involving greater incorporation of biodiversity into ecosystem function models. Pursuing both approaches will provide greater insight into biodiversity and ecosystem services projections. Integrating biodiversity, ecosystem function, and ecosystem service modeling will enhance policy development to meet global sustainability goals.

At present, global models of biodiversity and   ecosystem services estimate the impacts of anthropogenic stressors (e.g., climate change, land-use change) using scenarios that operate independently (Rosa et al. 2017). Projections of socioeconomic variables such as demography and land-use change are traditionally used to estimate impacts on biodiversity separately from those on ecosystem services (Pereira et al. 2010). Although efforts have been made to incorporate some attributes of nature or ecosystems (e.g., total biomass or particular service-providing species), these projections are often made without explicit consideration of the role of biological diversity itself in maintaining ecological functions underpinning the provision of ecosystem services (figure 1; Rosa et al. 2020), even though the scientific understanding of these links has advanced significantly (Isbell et al. 2017, van der Plas 2019). As a result, global projections of biodiversity, ecological functions, and ecosystem services may be overly optimistic because they assume that the remaining biological components of nature will continue to provide the same flow of benefits to people, regardless of how much biodiversity is lost (Isbell et al. 2015). For example, some carbon storage models assume constant carbon pools after land-use change (Kovacs et al. 2013), even though biodiversity loss continues over time after the initial habitat loss (Rosenzweig 1999). Efforts to integrate biodiversity and ecosystem function models are increasing but still fall short (Rosa et al. 2020).

**Figure 1.**
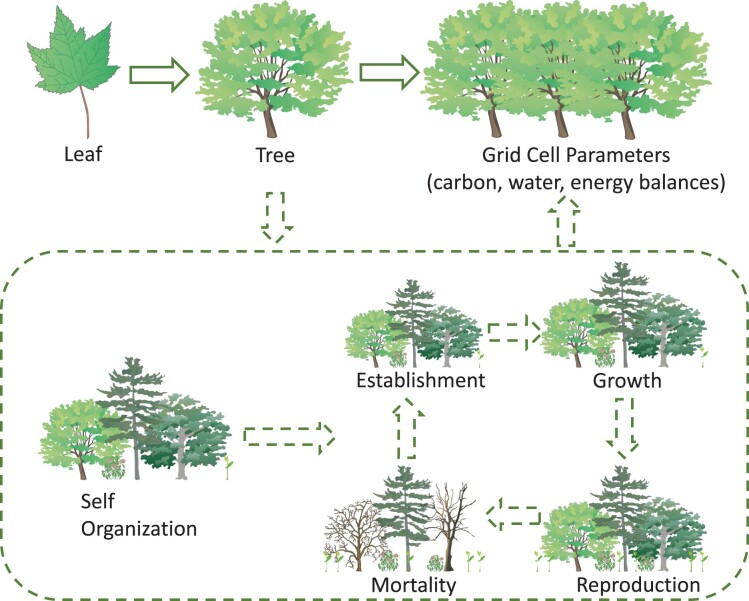
Many ecosystem function models do not incorporate the role of biological diversity in ecosystem function. For example, many traditional dynamic global vegetation models skip from leaves to individual trees to grid-cell parameters (the top panel) without incorporating detailed representation of demographic processes and vegetation composition (the dashed box and the arrows). The illustrations are courtesy of Tracey Saxby, Integration and Application Network (ian.umces.edu/media-library).

Many biodiversity and ecosystem function models operate at different spatial and temporal scales, which poses a challenge for integration (Isbell et al. 2017). Moreover, some models include parameters that do not incorporate biodiversity but that may be dependent on biodiversity (e.g., a carbon storage model may consider total biomass but not the species diversity that makes up that biomass). Therefore, before integration strategies can be developed, scientists need a synthetic understanding of the ways that existing models operate, including which biodiversity attributes and ecosystem functions are addressed, the inputs and outputs of each model type, and the spatial and temporal scales at which they work. This information will allow the scientific community to identify the areas of overlap between models and to determine which integration strategies are most feasible.

To review and develop strategies to link biodiversity and ecosystem service models, we convened a team of modelers and socioeconomic and policy experts as part of a SESYNC (National Socio-Environmental Synthesis Center) Pursuit project. We focused specifically on how to link biodiversity and ecosystem function models at the global scale, because this has been identified as a key knowledge gap and a first step toward full integration between biodiversity, ecosystem function, and ecosystem service modeling (Ferrier et al. 2016).

Specifically, the objectives of this study were to review existing biodiversity, ecosystem function, and ecosystem service models to identify areas of complementarity and mismatches; to develop a conceptual framework for how to link them to more fully account for the role of biological diversity in ecosystem functions; and to illustrate the potential of this framework through a case study. We restricted our review to models used in the intercomparison of biodiversity and ecosystem services models using harmonized scenarios (BES-SIM; Kim et al. 2018) and models from the Fisheries and Marine Ecosystem Model Intercomparison Project (Fish-MIP) intended to provide input to the Intergovernmental Panel on Climate Change (IPCC) sixth assessment report (see the supplemental material). Many of these models were used in the Intergovernmental Platform on Biodiversity and Ecosystem Services (IPBES) global assessment and the recent Bending the Curve initiative (Shin et al. 2019, Leclère et al. 2020). The choice of these models was driven by the intended audience of our model integration—namely, scientists (and, ultimately, policymakers) interested in tracking and investigating policy options for achieving the biodiversity-related Sustainable Development Goals and the Convention on Biological Diversity post-2020 biodiversity targets (United Nations 2015, Convention on Biological Diversity 2021).

## Model review

Using the two sets of global models used in recent model intercomparison projects (i.e., BES-SIM and FISH-MIP), we conducted a review to understand the possible links between the models that could reflect the increasing knowledge that biodiversity plays a role in many ecosystem processes. First, we assigned these models into three general categories: biodiversity models, ecosystem function models, and ecosystem service models (table 1). This approach follows the general breakdown of Kim and colleagues (2018) but splits the models assessing changes in ecosystem function from those that directly link to benefits to people (i.e., ecosystem services). Then, for each model, we extracted the following information: model inputs, model outputs, biodiversity metrics or ecosystem functions included or projected in the model, diversity-dependent parameters (if applicable), temporal scale, and spatial scale (resolution and extent). In the following paragraphs, we summarize information and attributes for the models included in our three model categories.

**Table 1.** Definitions for the three general categories of models included in the present article.

Model type	Definition
Biodiversity model	Models that project the current state of or the effect of environmental change on the biological components of ecosystems, such as genes, species, functional groups, and communities. Models commonly assess changes in species distribution, abundance, or community structure.
Ecosystem function model	Models that capture important ecological processes by modeling interactions between biotic and abiotic ecosystem components. Common processes captured in ecosystem function models include productivity, trophic interactions, and nutrient fluxes.
Ecosystem service model	Models that capture how changes in ecological conditions affect the goods and services that people receive from natural systems, usually resulting from one or more ecosystem functions. Common services captured by the models include provisioning services (e.g., timber) and regulating services (e.g., hydrology).

*Note:* Drawn from Ferrier and colleagues (2016). Distinctions between the model classes are not always clear, and some models can fit into multiple categories.

In total, we reviewed 29 models: 11 biodiversity models, 7 ecosystem function models, and 11 ecosystem service models, which we summarize in the following sections. Although a number of the models, especially the marine models, consider both ecosystem function and services, fewer of the models reviewed attempt to consider links (or the relationship between) biodiversity and function. The supplemental material provides the data extracted for each model considered in the review.

### Biodiversity models

**Figure 2.**
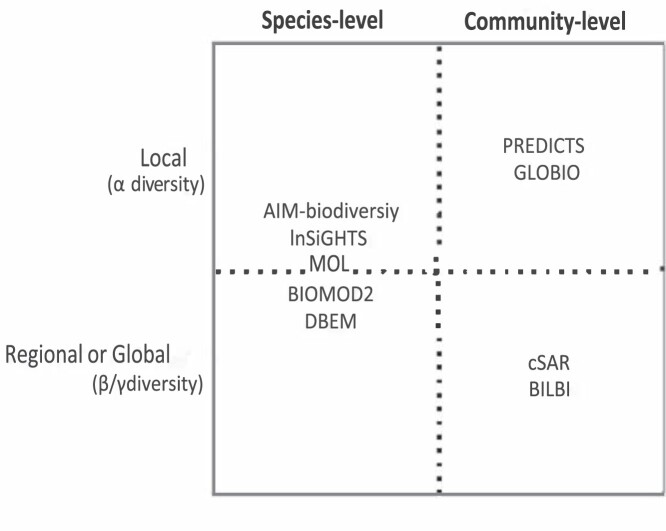
An overview of the correlative biodiversity models included in our review in terms of modeling approach (i.e., bottom-up species-level versus macroecological community level) and level of diversity assessed (i.e., changes in local diversity versus changes in collective regional or global diversity).

Of the 29 models reviewed, 11 were related to biodiversity measurements (10 mainly focused on terrestrial ecosystems and 1 on marine; figure 2). In general, the temporal scale was flexible, but an annual resolution was the most frequently used, depending on the environmental inputs needed by the model. For BES-SIM, all of the models were run up to 2050, but the temporal extent of the models can vary depending on user specifications. The spatial extent was flexible but usually global (global scale output was a requirement for BES-SIM), whereas the resolution (while also being flexible) varied greatly, from 50 meters to about 50 kilometers (see the supplemental material).

Most of the biodiversity models were correlative models. We further classified these correlative biodiversity models on the basis of modeling approach and scale (figure 2). Some of the models use a bottom-up approach, in which species-level models are layered to arrive at overall biodiversity estimates. These models can provide alpha (local), beta (regional species turnover), or gamma (overall regional or global) diversity estimates across the spatial extent over which the models are run (e.g., an ecoregion, or a major ecosystem type). Other models use a macroecological approach, in which changes in drivers are used to predict changes in total alpha, beta, or gamma diversity in a top-down manner, without explicitly modeling changes in individual species.

Some of the models included in our review cannot be neatly categorized into either biodiversity or ecosystem function or service models. Several process-based models encode some aspects of biodiversity. For example, the Madingley model is a process-based, mechanistic general ecosystem model that represents cohort dynamics for heterotrophic organisms and stocks of autotrophic organisms for both terrestrial and marine ecosystems (Harfoot et al. 2014). Although some level of diversity is included in the model, there is room to expand the level of diversity represented.

### Ecosystem function models

We classified seven models as ecosystem function models, primarily on the basis of the main model output (see table 1). For example, the model outputs included species biomass and production. Because we reviewed these models in the context of IPBES and IPCC analyses, the spatial extent tended to be global, but some models also made predictions constrained to regional and local scales. The temporal resolution of the ecosystem function models varied from static predictions to hourly, daily, monthly, quarterly, and yearly predictions (see the supplemental material).

### Ecosystem service models

We classified 11 models as ecosystem service models, some of which had submodules (including InVEST and GLOBIO). The key element that distinguished ecosystem service models from the model types discussed above is that they had an explicit connection to how a change in the ecosystem led to changes in ecological contributions to humans (or even expression of this change in monetary values). The spatial resolution of these models varied widely, ranging from half degree at the coarsest level to 10 meters at the finest scale. Temporal scale was flexible for most models and determined by the frequency of input data (see the supplemental material).

### Biodiversity-dependent model parameters

As part of our review, we identified a number of biodiversity-dependent parameters in the ecosystem function and services models that could be used to explicitly link these models to biodiversity models (see the supplemental material). For example, the InVEST crop pollination model contains several input and output parameters dependent on biodiversity, such as floral resources, foraging activity, bee relative abundance, and wild pollinator yield. Other examples include wild food and protection against erosion output parameters in the GLOBIO ecosystem services model and an array of output parameters (e.g., production rates, habitat extent, catch) from Atlantis. Likewise, for the process-based models (e.g., dynamic global vegetation models), we assessed which mechanisms could be improved to better reflect the role biodiversity plays in determining changes in functions.

## Model integration strategies

**Figure 3.**
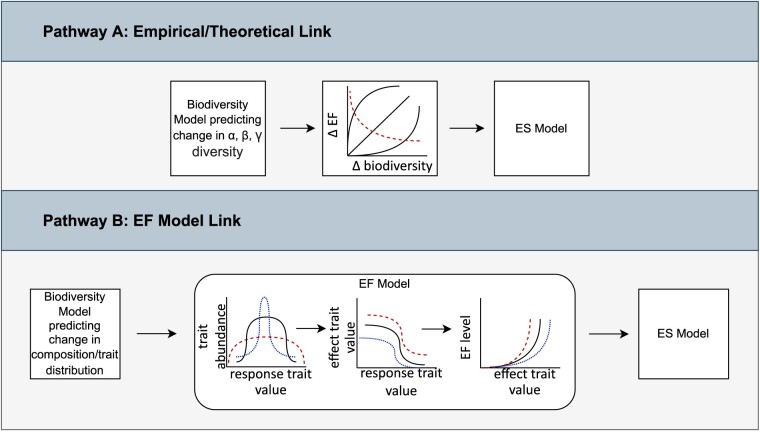
Conceptual framework for integrating biodiversity, ecosystem function (EF), and ecosystem service (ES) models. Pathway A links community biodiversity models that predict changes in overall *a*, *β*, or *γ* diversity to ecosystem service models using established biodiversity–ecosystem function relationships derived from empirical data. The dashed line in the center panel indicates that in some cases, biodiversity can decrease ecosystem function. Note that Δ biodiversity indicates that biodiversity could either increase or decrease from its current state. In pathway B, species or trait-based biodiversity models can be used to set initial conditions for trait-based ecosystem function models. Differential survival of functional groups may lead to changes in the distribution of effect traits (i.e., traits that influence ecosystem properties). Ideally, one could capture biodiversity and ecosystem function relationships within the EF model itself as well; for instance, communities where a stressor (e.g., climate or land-use change) greatly reduced initial response-trait diversity (i.e., traits that influence species’ ability to persist in the face of environmental change; the blue-dotted line) would have reduced function even at the same level of response trait than communities starting out with higher levels of response trait diversity (the black solid and red dashed lines).

After conducting the review and identifying potential integration links, we developed a novel conceptual framework for model integration, in which we identify two possible pathways for achieving such integration (figure 3). The first uses empirical data on biodiversity-ecosystem function relationships to link biodiversity model output directly to ecosystem services models. The second uses ecosystem function models to link biodiversity and ecosystem services. For each pathway, we aimed to address four issues: What data are needed to allow the integration? What future research is needed to be able to implement these links? Which ecosystem functions would be best suited for each of these pathways? And which models from the existing set would be best suited for each pathway?

### Pathway A: Using biodiversity–ecosystem function relationships derived from empirical data to bridge biodiversity and ecosystem service models

One way to account for the dependence of ecosystem services on changes in biodiversity is to use established biodiversity–ecosystem function relationships derived from empirical data to bridge models that project how biodiversity will change with models that project how ecosystem services will change. Doing so requires identifying biodiversity models with outputs and ecosystem service models with inputs that correspond to well-studied empirical relationships between biodiversity and ecosystem function.

Anthropogenic effects on the biosphere have effectively provided a large-scale experiment on what happens when you radically change both species richness and composition. There are numerous biodiversity assessments (e.g., Díaz et al. 2019) and a rich literature on the effects of cumulative change on the world's ecosystems (such as the effects of fishing down or through food webs; Pauly et al. 1998, Essington et al. 2006). Although these observations can be used to validate the broadscale predictions made by the models, they often do not allow for disentangling the extent to which any observed change in ecosystem function is due to change in the number of species (richness), which species are present (composition), or both.

Biodiversity experiments are, however, designed with this in mind and provide an avenue for informing integration. For instance, hundreds of biodiversity experiments have manipulated plant species richness and measured effects on plant productivity (O'Connor et al. 2017, 2022). Therefore, biodiversity models that project future changes in plant species richness and ecosystem service models that depend on plant productivity would be good candidates for integration aimed at addressing this relationship.

Many experiments on the relationship between biodiversity and ecosystem function assess changes in productivity due to changes in species richness, and meta-analyses of biodiversity experiments have produced estimates of how changes in species richness lead to overall biomass changes (e.g., O'Connor et al. 2017). As such, biodiversity models used in this pathway could assess how projected changes in drivers (e.g., climate change or land-use change) lead to changes in plant species richness. Many correlative biodiversity models can produce these estimates, including bottom-up species models that are stacked to produce estimates of species richness changes, as well as top-down community-level, or macroecological, models that produce estimates of changes in alpha, beta, and gamma diversity without considering specific species (figure 2). The resulting output could be converted to proportional change in species richness.

Using the aforementioned relationships between species richness and productivity, altered plant productivity output could then be fed into an ecosystem service model. For example, the InVEST model (Natural Capital Project 2022) requires users to upload a table with carbon storage values for different land-use types. Modelers could use the expected productivity changes to update the carbon storage values for different land-use types on the basis of the expected biodiversity loss.

Early steps have already been taken to implement this approach. For example, Isbell and colleagues (2015) linked estimated species extinction debts associated with land-use change with biodiversity and ecosystem function relationships obtained from empirical and theoretical studies, such that

F = 1 – {1 – D)ab,

where *F* represents ecosystem function debt, *D* represents the proportion of habitat destroyed, *α* is a constant indicating the magnitude of the extinction debt, and *β* is a constant indicating the strength of the biodiversity and ecosystem function relationship. They then linked this with an ecological production function for carbon, and used existing land-use and global biomass maps to estimate the gradual carbon loss in remaining habitat fragments expected to result from past land-use changes.

Using another approach, Mori and colleagues (2021) created species distribution models for tree and shrub species globally under present and projected future conditions and combined this with multiple modeling methods to obtain changes in species richness between 2005 and 2070. They converted this change in species richness to changes in proportional forest productivity using elasticity of substitution values (i.e., the degree to which species can substitute for each other in contributing to stand productivity; Liang et al. 2016) estimated from forest inventory data sets. By comparing different climate change scenarios, they were able to estimate the mitigation value of conserving forest plant diversity in different regions and globally.

Although we highlight plants and carbon storage, other taxa and services are also suitable for this method where sufficient understanding of biodiversity–ecosystem function relationships exist. However, the kinds of intentional experiments needed have most commonly been undertaken for plants, especially in temperate grasslands, and there are fewer such experiments for other taxa or in the marine realm. Consequently, it may be necessary to draw the relationships on the basis of spatial or temporal gradients of depletion (noting the potential for uncertainty injected by confounding processes).

### Pathway A: Assumptions and challenges

When using data from biodiversity experiments, it is important to carefully consider several aspects of their study designs. First, most biodiversity experiments were designed to disentangle effects of species richness, per se, from those of species identity—for example, the importance of legumes in terrestrial systems (Spehn et al. 2002) or whales in marine systems (Roman et al. 2014). When a species is lost from an ecosystem, two things change simultaneously: Species richness decreases, and species composition shifts. Biodiversity experiments have rigorously quantified the effects of richness and composition by randomizing both how many and which species are included in each experimental plot (Tilman et al. 2014). This creates a gradient of plant species richness without systematically changing which species are present at each level of plant diversity. Therefore, variation in ecosystem function *between* experimental levels of richness indicates an effect of plant diversity that is independent of any effects of changing which species are present. Variation in ecosystem function *within* an experimental level of richness indicates and effect of species composition that is independent of any effects of species richness. Richness and composition effects can be equally strong (Hector et al. 2011). In natural ecosystems, it will often be important to account for the effects of changes in both richness and composition, which may be reinforcing (i.e., if the most productive species are systematically lost) or counterbalancing (i.e., if the least productive species are systematically lost; Smith and Knapp 2003, Isbell et al. 2008). This is easier said than done. Trait-based frameworks seek to predict which types of species will tend to be lost or favored under future environmental conditions. To the extent that these traits can be identified and rigorously linked to both global changes and ecosystem services, trait-based approaches may help provide information for composition effects (see pathway B below).

Second, positive effects of biodiversity on ecosystem function are often observed in natural systems, where species composition is not manipulated (e.g., Liang et al. 2016). However, the strength and direction of biodiversity and ecosystem function relationships can differ across ecosystem functions and ecosystem types in natural communities (van der Plas 2019). Moreover, the number of species included in local-scale experiments tends to be much lower than the diversity of natural communities, and therefore, the proportional loss of function with loss of species richness may not be directly transferable (Mori et al. 2016, Manning et al. 2019, but see Jochum et al. 2020). This and other uncertainties in the current knowledge that stems from the local-scale experiments could add a considerable amount of error to model predictions for the consequences of biodiversity loss on ecosystem function. Model intercomparison efforts in the biodiversity and ecosystem function literature (e.g., Crawford et al. 2021) can help explore differences across systems and improve the generalizability of biodiversity and ecosystem function relationships.

Finally, biodiversity and ecosystem function relationships are likely to be scale dependent (Barry et al. 2021), and there are often mismatches between the scale of biodiversity and ecosystem function experiments and the scale of biodiversity model outputs (Isbell et al. 2017). Biodiversity can only be experimentally manipulated at relatively small, local scales, which means that the results from biodiversity experiments do not yet account for additional effects of biodiversity that theory predicts can arise at larger spatial scales. Therefore, biodiversity experiments have rigorously considered how local species interactions can lead to effects of biodiversity on ecosystem function but have not yet been able to account for processes, such as dispersal, which can create additional effects of biodiversity on productivity at larger spatial scales. This may be especially important, because species richness and species turnover are not always coupled and are, therefore, not on their own sufficient for capturing biodiversity changes (Blowes et al. 2019).

At local scales, increasing plant diversity can increase productivity by reducing competition, increasing facilitation, or both (Hooper et al. 2005). At larger landscape scales, dispersal, which is controlled and not considered in local biodiversity experiments, can regulate both biodiversity and productivity. In many meta-community models, at low rates of dispersal, species fail to reach the parts of the landscape where they would be most productive (Loreau et al. 2003). This can limit both biodiversity and productivity across the landscape. At excessively high dispersal rates, the single species that is the best competitor for the average conditions across the entire landscape can drown out all other species, again leading to relatively low diversity and low productivity, given that some species that would have otherwise been more productive at some places in the landscape are outcompeted by a less productive species (Loreau et al. 2003). At intermediate dispersal rates, species can optimally sort across heterogeneous environments, leading to relatively high levels of diversity, with species reaching and becoming dominant in the parts of the landscape where they are most productive (Loreau et al. 2003). This could create an effect of spatial beta diversity (i.e., turnover in species from one place to another) on productivity that is usually not accounted for in local biodiversity experiments. Therefore, when using results from biodiversity experiments to bridge these relationships, it is important to keep in mind that they likely include some, but not all, relevant effects of biodiversity on ecosystem function. Moving forward, it may be possible to incorporate both experimental data and theoretical scaling relationships (e.g., Gonzalez et al. 2020) in models that project how changes in beta diversity may affect ecosystem function and ecosystem multifunctionality (Pasari et al. 2013). This will allow for greater assessment of how processes such as homogenization of communities across scales may affect ecosystem function over time.

### Pathway B: Using biodiversity model outputs to parameterize models that predict changes in ecosystem function

Trait-based models have proven to be a particularly effective means of modeling ecosystems, with different types of models focusing on different key traits. For example, size has a strong influence on many ecological processes—metabolic strategies and costs, sensory and feeding types, mobility and other life history strategies (Andersen et al. 2016)—and so can be a particularly useful shorthand for modeling ecosystem composition and how it may respond to stressors (Blanchard et al. 2017). This is true both in marine and terrestrial ecosystems (e.g., Brown et al. 2004, Brose et al. 2006). Although functional traits underpin an organism's contribution to ecosystem function and its tolerance to environmental pressures (e.g., (Suding et al. 2008), the contributions and responses of an organism will often depend on the combinations and tradeoffs of traits rather than any individual trait (e.g., Díaz et al. 2013).

Given the influence of traits on ecosystem function and services and how individual species represent specific trait combinations, traits are a logical choice for integrating biological diversity (via trait composition) into ecosystem function models. Over more than 40 years, marine scientists have used ecosystem models built around key species or functional groups to try to understand ecosystem function and predict the outcomes of changing drivers (e.g., climate change driven temperature shifts, changing ocean acidification levels, or fishing pressure; Christensen and Walters 2004, Blanchard et al. 2012).

Similarly, for terrestrial ecosystems, there is a long history of trait-based models for vegetation in which traits specific to plant functional types are used to simulate their physiology, demography, and ecological dynamics (Scheiter et al. 2013, Fisher et al. 2018). More recently, approaches analogous to marine ecosystem models have been developed and applied to simulate the functional composition and ecosystem function of whole terrestrial ecosystems (Harfoot et al. 2014). Consequently, it is a conceptually straightforward step to consider how to exploit those model structures to more thoughtfully include biological diversity. Although the reality of such implementation is complicated (given the many thousands of species present in most ecosystems), we identify three potentially fruitful modeling strategies.

**Figure 4.**
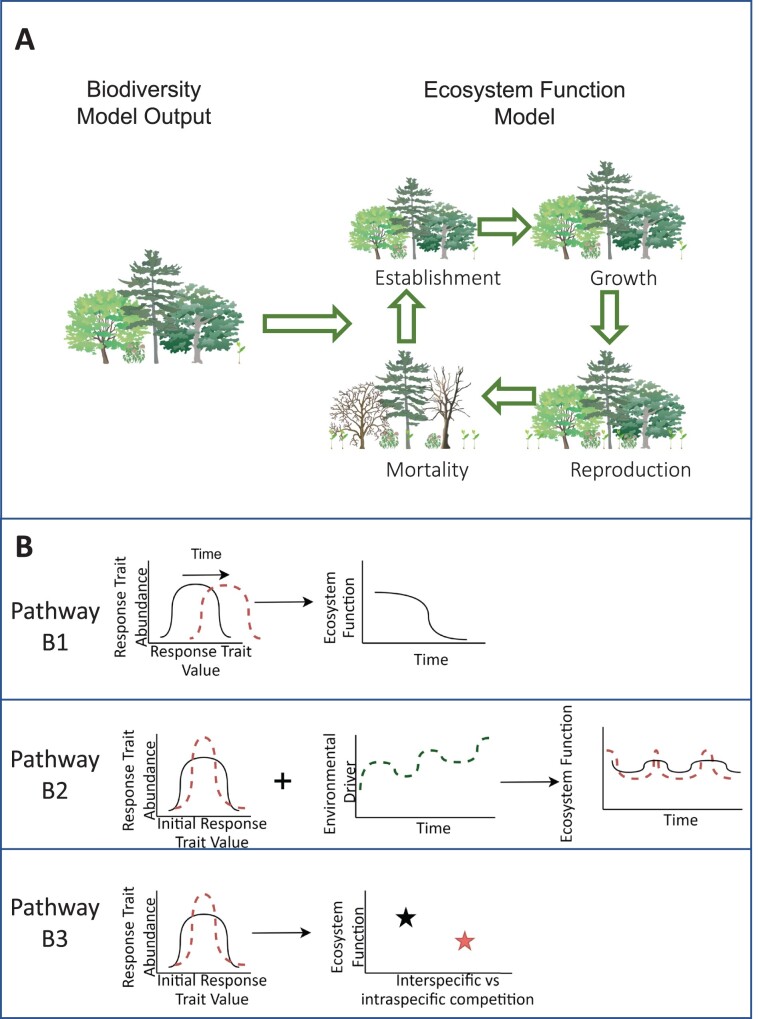
Example pathway B integration strategies for a vegetation ecosystem function model that incorporates vegetation dynamics. Panel (a) shows how biodiversity model output can parameterize an ecosystem function model. Panel (b) illustrates different ways that ecosystem function models can use trait information from biodiversity models to project changes in ecosystem function. In pathway B1, a biodiversity model is used to set the initial response trait distribution of the system (the solid line in the left graph). As the vegetation model runs, the response trait distribution will change over time (the red dashed line), leading to changes in ecosystem function (the solid line in the right graph). In pathway B2, the model considers not only the initial trait distribution, but also trait variation. As environmental conditions change over time (the dashed line in the middle graph), systems with greater variance in the trait distribution (the solid line as compared with the dashed line in the left graph) may have more stable ecosystem function over time (the solid line as compared with the dashed line in the right graph). In pathway B3, ecosystem function models also consider mechanistic drivers of biodiversity–ecosystem function relationships. For example, more diverse systems (the solid line in the left graph) may have higher ecosystem function if interspecific competition is lower than intraspecific competition (the black star in the right graph). Some models may use a hybrid of these three approaches. The illustrations in panel (a) appear courtesy of Tracey Saxby, Integration and Application Network (ian.umces.edu/media-library).

The simplest approach (figure 4, pathway B1) is simply to mimic the outcome of differential drivers on the activities or survival of functional groups, with different combinations of response traits (i.e., traits that influence species’ ability to persist in the face of environmental change), such as body size, trophic strategy, and thermal tolerance. Differential survival of these functional groups may lead to changes in the distribution of effect traits (i.e., traits that influence ecosystem properties) in the ecosystem and, therefore, the ecosystem function and services provided (Díaz et al. 2013). Early steps have been taken in this direction via explicit attempts to model evolution within food webs (e.g., Forestier et al. 2020), especially under climate change and how that may affect regional fisheries (e.g., Fulton and Gorton 2014). However, experience to date indicates that such approaches are likely insufficient for authentically capturing the dynamic outcomes of interactions between ecosystem function, species composition, and the different forms of biodiversity (Fulton et al. 2019). Instead, integration is needed across processes and scales relevant to both ecosystem function and species or trait composition (Mokany et al. 2016, Grimm et al. 2017). This may require linking (at least conceptually) across model types.

Another approach (figure 4, pathway B2) is to use existing species-level biodiversity models (figure 2) to provide information on the species present in a particular ecosystem that could be grouped to comprise a functional group. This then informs the functional groups and initial distribution of traits that will be included in the model. Some classes of models already represent trait distributions within functional groups, most notably marine size spectra models (Blanchard et al. 2017). However, other functional group-based models represent organisms using a central estimate of trait values. For example, in the Madingley general ecosystem model, the cohorts of the model, used to represent collections of individuals belonging to a categorical functional group, differ in trait values across cohorts, but within a cohort, all individuals are identical (Harfoot et al. 2014). To address this, functional group-based models could adopt moment-based approaches (Norberg et al. 2001) to parsimoniously represent trait distributions and how ecological processes affect these. Doing so could allow models to capture the biodiversity as insurance hypothesis; that is, because species or functional groups respond differently to environmental changes, greater diversity promotes greater stability of functions over time (Yachi and Loreau 1999).

Finally, a third approach (figure 4, pathway B3) would be to use theoretical understanding of biodiversity and ecosystem function relationships to inform processes in an ecosystem function model. As was discussed above, there is strong evidence that complementarity between species can lead to increased function at higher levels of diversity, and these effects grow stronger over time (Reich et al. 2012). In terrestrial systems, vegetation demographic models that explicitly represent demographic processes and individual-based competition have recently been developed (Fisher et al. 2018), allowing for more realistic simulations to investigate how biodiversity and community assemblage changes in the course of vegetation succession might lead to changes in vegetation composition, biogeochemical cycles, and productivity. Modeling reduced intraspecific competition at higher diversity levels or selection for more functionally distinct species over time could be one way to capture complementarity effects.

### Pathway B: Assumptions and challenges

One assumption of these methods is that functional groups accurately represent the diversity present in ecosystems. More realistically representing diversity within ecosystem function models is challenging. For example, in many dynamic global vegetation models, the biodiversity of vegetation is simplified into approximately 10–15 plant functional types across the globe (Sitch et al. 2003). Even for site-level simulations, the vegetation community is usually represented by only a few dominant species (e.g., Purves et al. 2008). Moreover, there can be strong effects of changing diversity even *within* functional groups (Reich et al. 2004). A major obstacle for improving functional diversity in ecosystem function models is computational demand. Another challenge is incorporating the relevant processes that determine how biodiversity responds to drivers of change; this is because most models do not include sufficient representation of small-scale processes that introduce a degree of density dependence and there is a tendency for model self-simplification (i.e., loss of biodiversity as the model is run).

Another key assumption in this pathway is that we accurately capture the relationship between response and effect traits. The composition of species in an ecosystem is driven by how species respond to changes in the environment (i.e., response traits), but this may or may not be related to their ability to provide specific ecosystem functions (Díaz et al. 2013). Even when traits are linked, species respond to multiple drivers simultaneously, which may act on species in different ways, potentially complicating the links between response and effect traits and, therefore, the optimal combination of traits. In addition, we lack an understanding of how global changes will affect trait distributions. For example, will climate change favor types of plants that are more or less productive, and will the same or different types of plants be productive under future environmental conditions? Things become even more complex when species interactions further complicate outcomes via differential effects of changes in predation, competition, parasitism, and mutualism on species productivity and abundance. Attempting to incorporate these relationships may therefore lead to greater uncertainty around estimates of ecosystem function change, but this uncertainty could be reduced over time as knowledge and data improve.

Finally, limitations in our theoretical understanding of biodiversity and ecosystem function relationships can make incorporating them into process-based models difficult. For example, complementarity can be driven by multiple processes, including resource partitioning, abiotic facilitation, and biotic feedbacks (Barry et al. 2019). Many studies measure complementarity effects without considering the underlying mechanism, whereas process-based models incorporate mechanisms and then model the resulting effects on function (Barry et al. 2019). Therefore, more mechanistic theoretical work may be needed before these concepts can be fully incorporated into process-based models. On the other hand, process-based models may be useful tools to explore the consequences of different biodiversity and ecosystem function mechanisms (see box 1).

**Box 1. Case study: A simulation of biodiversity–productivity relationships using forest crown organization and community light use efficiency.**

Light environment is a key factor regulating plant species competition (Aubin et al. 2000, Bartemucci et al. 2006). High biodiversity can increase light interception by filling canopy gaps, increasing ecosystem productivity (Pretzsch 2014, Duarte et al. 2021, Williams et al. 2021). Early successional species usually have higher photosynthesis, growth, and mortality rates and high crown gaps compared with late successional species (Pacala et al. 1996, Caspersen and Pacala 2001). In the present article, we conceptually illustrate how pathway B3 could be used to reproduce this pattern, and how models could be modified to illustrate pathways B1 and B2 (figure 4). Our analysis has unresolved assumptions, but is meant to show how more complex models could be applied.

We used a demographic vegetation model, BiomeE (Weng et al. 2019), to simulate patterns of biodiversity-mediated changes in community light interception and productivity in forest succession. BiomeE assumes crowns can be any shape to fill space and the light competition is simulated as a function of tree height and crown area. In this test, we used two parameters to represent tradeoffs between light interception and plant physiology—the fraction of canopy intrinsic gap (*f*_gap,0_) and the maximum rate of carboxylation (*V*_cmax_). *f*_gap,0_ affects leaf distribution and light penetration through canopy layers. *V*_cmax_ defines photosynthesis and respiration rates at given light and temperature conditions and, therefore, a tree's shade tolerance. Early successional species usually have a high *V*_cmax_, a low leaf area index, and high canopy gaps. Late successional species usually have a low *V*_cmax_, a high leaf area index, and low canopy gaps. We defined two plant functional types (PFTs; supplemental table S2): an early successional, shade-intolerant PFT that has a high *f*_gap,0_ and *V*_cmax_ and a late successional, shade-tolerant PFT that has a low *f*_gap,0_ and *V*_cmax_. We conducted monoculture and polyculture simulations (supplemental table S3). In the polyculture run, the fraction of canopy gaps was calculated as a function of relative species abundances, accounting for possible resource partitioning using the following equation: 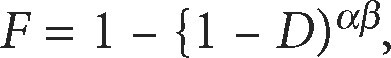,

where *f_gap0,i_* is the default gap fraction of PFT *i*, *p_i_* is the fraction of crown area of PFT *i* in the total crown area. For monoculture runs, *f*_gap_ is always equal to *f*_gap0_.

**Figure 5.**
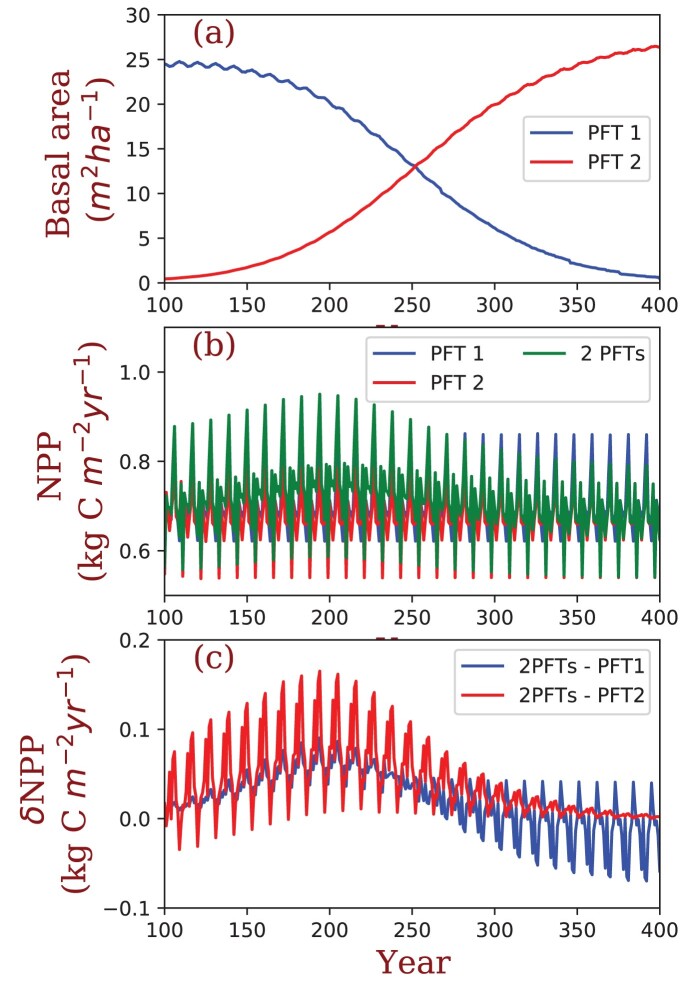
BiomeE simulations exploring the relationship between biodiversity and net primary productivity (NPP). (a) As the model progresses, the early successional species PFT1 is gradually replaced by PFT2. (b) When PFT1 and PFT2 are both present in the model, productivity is higher than PFT1 or PFT2 alone. (c) Difference in NPP (dNPP) between the polyculture run and monoculture runs. Values above 0 indicate that productivity is higher in the polyculture run.

Succession in the polyculture run generates a biodiversity gradient through time, with the dominant species shifting from PFT1 to PFT2 (figure 5a). Net primary productivity (NPP) increased when both PFT1 and PFT2 were in the community (figure 5b and 5c) and was higher than NPP of the monoculture runs. This indicates gap filling with high biodiversity may be a key mechanism increasing light use efficiency and NPP, but data to parameterize and validate the model are needed before we can draw conclusions.

**Next steps to fully implement pathways B1–B3**

In this example, we artificially selected trait parameters for each PFT, but in the future, biodiversity models could be used to determine initial trait values (pathway B1). A few modifications could be made to implement pathway B2: In addition to setting a mean trait value for each PFT, one could assign variance around the mean. Alternatively, one could include an environmental driver that changes over time that results in changes in optimal trait values. Functional groups with greater trait variance may have lower productivity under certain values of the environmental driver (i.e., not all individuals have the optimal trait value at any given time), but may respond to changes more quickly. In all pathways, additional PFTs could be added to further explore the effects of biodiversity on ecosystem function.

## Conclusions

Integrating biodiversity and ecosystem function ­models will improve our ability to predict how changes in drivers will lead to changes in important ecosystem services, which will be critical for developing policies to meet the goals of the post-2020 global biodiversity framework and the Sustainable Development Goals. In the present article, we have proposed two distinct pathways for integrating biodiversity and ecosystem function models. Pathway A, which uses empirical data on biodiversity–ecosystem function relationships to bridge biodiversity and ecosystem function models, provides a feasible strategy that could currently be implemented at the global scale for systems and taxa with sufficient data. In addition to this low-hanging fruit approach, we also propose pathway B, a trait-based strategy involving greater incorporation of biological diversity into existing ecosystem function models that can be applied to more systems and taxa than pathway A. We argue that pursuing both approaches simultaneously will provide greater insight into projections of biodiversity and ­ecosystem services.

Using multiple strategies and models to predict how changes in biodiversity could lead to changes in ecosystem function may enhance the uptake of information in decision-making and policy discussions by increasing confidence in model results and delivering more relevant information. Comparing the results obtained for the same process using multiple techniques will allow us to better understand uncertainty and, in cases of model agreement, improve confidence in outputs. This is similar to other ongoing large model intercomparison projects (e.g., CMIP, BES-SIM, FISH-MIP). Using multiple approaches will also expand the list of model outputs, thereby increasing the likelihood that results will be relevant and engage policymakers’ interests.

In addition to improving projections of biodiversity and ecosystem services, the proposed model integration approaches can be used to improve our understanding of biodiversity and ecosystem function relationships in general. For example, much of our understanding of biodiversity and ecosystem function relationships comes from small-scale field experiments that are difficult to test at large spatial scales and are only possible for a limited set of taxa. Process-based models employed in pathway B provide an opportunity to run simulations at large spatial scales and for more taxa, thereby improving our understanding of how biodiversity and ecosystem function relationships scale. In particular, improving our understanding of how beta diversity affects functions (e.g., by allowing for colonization from nearby areas) would be particularly useful. Conversely, empirical data on biodiversity and ecosystem function relationships can be used to improve understanding of the links between response and effect traits included in process-based models and also to validate expected results.

In the sections above, we highlighted important knowledge gaps for implementing our proposed model integration approaches. Addressing these gaps will improve our ability to accurately reflect the role of biological diversity in ecosystem functions and services. Despite the challenges of doing so, it is important to begin building the modeling infrastructure to integrate biodiversity and ecosystem function models. Once developed, models can be periodically updated to reflect improved understanding of biodiversity and ecosystem function relationships.

## Supplementary Material

biac074_Supplemental_Files

## Author Biographical

Sarah R. Weiskopf is affiliated with the US Geological Survey National Climate Adaptation Science Center, in Reston, Virginia, in the United States. Bonnie J. E. Myers is affiliated with the North Carolina Cooperative Fish and Wildlife Research Unit, in the Department of Applied Ecology at North Carolina State University, in Raleigh, North Carolina, in the United States. Maria Isabel Arce-Plata and María Cecilia Londoño-Murcia are affiliated with the Instituto de Investigación de Recursos Biológicos Alexander von Humboldt, in Bogotá, Colombia. Julia L. Blanchard is affiliated with the Institute for Marine and Antarctic Studies and with the Centre for Marine Socioecology at the University of Tasmania, in Hobart, Tasmania, Australia. Simon Ferrier is affiliated with Land and Water, CSIRO, in Canberra, in the Australian Capital Territory, Australia. Elizabeth A. Fulton is affiliated with Oceans and Atmosphere, CSIRO, and with the Centre for Marine Socioecology at the University of Tasmania, in Hobart, Tasmania, Australia. Mike Harfoot is affiliated with the United Nations Environment Programme–World Conservation Monitoring Centre, in Cambridge, England, in the United Kingdom and with Vizzuality, inMadrid, Spain. Forest Isbell is affiliated with the Department of Ecology, Evolution, and Behavior at the University of Minnesota, in Saint Paul, Minnesota, in the United States. Justin A. Johnson is affiliated with the Department of Applied Economics at the University of Minnesota, in Saint Paul, Minnesota, in the United States. Akira S. Mori is affiliated with the Research Center for Advanced Science and Technology at the University of Tokyo, in Meguro, Tokyo, Japan. Ensheng Weng is affiliated with the Center for Climate Systems Research at Columbia University and with the NASA Goddard Institute for Space Studies, both in New York, New York, in the United States. Zuzana V. Harmáčková is affiliated with the Global Change Research Institute of the Czech Academy of Sciences, in Brno, Czechia and with the Stockholm Resilience Centre, at Stockholm University, in Stockholm, Sweden. Brian W. Miller is affiliated with the US Geological Survey North Central Climate Adaptation Science Center, in Boulder, Colorado, in the United States. Laura M. Pereira is affiliated with the Global Change Institute at the University of the Witwatersrand, in Johannesburg, South Africa and with the Stockholm Resilience Centre, at Stockholm University, in Stockholm, Sweden. Isabel M. D. Rosa is affiliated with the School of Natural Sciences at Bangor University, in Gwynedd, in Wales, in theUnited Kingdom.
