# Supplementary material for: A Conceptual Framework to Integrate Biodiversity, Ecosystem Function, and Ecosystem Service Models
Source: Bioscience. 2022 Sep 1;72(11):1062–73. doi: 10.1093/biosci/biac074 (PMC9718641; doi:10.1093/biosci/biac074)
Supplement: biac074_Supplemental_Files [file biac074_supplemental_files.zip › Supplemental_tables_2_and_3.docx]

Supplemental **Table 2: Simulation Model Run Parameters**

| Parameter | PFT 1  (Early successional) | PFT 2  (Late successional) |
| --- | --- | --- |
| *V*_cmax_ (m mol m^-2^ s^-1^) | 20.0E10-6 | 12.0E-6 |
| *f*_gap0_ (fraction of canopy gaps) | 0.2 | 0.1 |
| Maximum crown LAI | 3.0 | 4.0 |
| Canopy mortality rate | 0.04 | 0.02 |
| Understory mortality rate | 0.1 | 0.5 |

**Supplemental Table 3: Simulation experiments**

| **Experiment** | **Setting** | **Runs** |
| --- | --- | --- |
| Monoculture | PFT: 1 or 2  *f*_gap_: 0.1, 0.2 for PFT 1 and 2, respectively  Initial density: 1000/ha for each run | 2 |
| Polyculture | PFTs: 1 and 2  *f*_gap_: change with the crown area ratio of PFT1 and PFT2 during the model run following Eq. 1.  Initial density: 1000/ha (PFT1) and 100/ha (PFT 2) | 1 |
