# Supplementary material for: A Conceptual Framework to Integrate Biodiversity, Ecosystem Function, and Ecosystem Service Models
Source: Bioscience. 2022 Sep 1;72(11):1062–73. doi: 10.1093/biosci/biac074 (PMC9718641; doi:10.1093/biosci/biac074)
Supplement: biac074_Supplemental_Files [file biac074_supplemental_files.zip › Supplement_1.pdf]

Metadata

Variable	Description	Type
**Basic Model Info Tab**		
Model Name	Name of model	open-ended
Source	Source of model info	open-ended
Model Description	A brief description of the model	open-ended
Model Focus	Note whether model is a biodiversity model, an ecosystem function model, or an ecosystem service model	CODED
Temporal Scale	Time frame of model output	open-ended
Spatial Scale (resolution)	Spatial resolution of model output	open-ended
Spatial Scale (extent)	Spatial extent of model output	open-ended
**Model Details Tab**		
Model Name	Name of model	open-ended
Variable Name	Name of variable included in model input or output.	open-ended
Variable description	Detailed description of the variable	open-ended
Input/Output	Is this variable a model input or model output?	CODED
Variable Class	Note whether variable is a biodiversity, ecosystem function (EF), or ecosystem service (ES) metric. Other options include pressure variables (drivers of change in biodiversity, EF, ES variables), habitat (species habitat preferences), or economic (economic valuation estimates)	CODED
Biodiversity Dependent Variable?	Is the variable one that does not currently incorporate biodiversity but that may be biodiversity dependent? Only relevant to EF and ES models	CODED

Basic Model Info

Model Name	Source	Model Description	Model Focus	Temporal Scale	Spatial Scale (resolution)	Spatial Scale (extent)
AIM-biodiversity (Asia-Pacific Integrated Model-biodiversity)	Ohashi, H., T. Hasegawa, A. Hirata, S. Fujimori, K. Takahashi, I. Tsuyama, K. Nakao, Y. Kominami, N. Tanaka, Y. Hijioka, and T. Matsui. 2019. Biodiversity can benefit from climate stabilization despite adverse side effects of land-based mitigation. Nature Communications 10(1):1–11.	Predicts potential shifts of suitable habitat of multiple species under multiple projected climate and land-use change scenarios. Model run using Maxent, using GBIF data for species occurrences. Taxonomic dispersal abilities considered in the model.	a. biodiversity model	2005, 2050, 2070	0.5 arc degrees	Global
InSiGHTS	Rondinini et al., 2011. Global habitat suitability models of terrestrial mammals, Philos. Trans. R. Soc. B Biol. Sci., 366(1578), 2633–2641, doi:10.1098/rstb.2011.0113, 2011.<br><br>Visconti, P., Bakkenes, M., Baisero, D., Brooks, T., Butchart, S. H. M., Joppa, L., Alkemade, R., Di Marco, M., Santini, L., Hoffmann, M., Maiorano, L., Pressey, R. L., Arponen, A., Boitani, L., Reside, A. E., van Vuuren, D. P. and Rondinini, C.: Projecting Global Biodiversity Indicators under Future Development Scenarios: Projecting biodiversity indicators, Conserv. 15 Lett., 9(1), 5–13,doi:10.1111/conl.12159, 2016.	High resolution species-specific habitat suitability models for the terrestrial mammals based on the IUCN Red List, to estimate the extent of suitable habitat for each species	a. biodiversity model	2010, 2050	300m	Global
MOL (Map of Life)	Jetz, W., Wilcove, D. S., Dobson, A. P., and Mace, G. M. (Eds.): Projected Impacts of Climate and Land-Use Change on the Global Diversity of Birds, PLoS Biol., 5, e157, https://doi.org/10.1371/journal.pbio.0050157, 2007.<br><br>Merow, C., Smith, M. J., and Silander, J. A.: A practical guide to MaxEnt for modeling species' distributions: what it does,<br>and why inputs and settings matter, Ecography, 36, 1058–1069, https://doi.org/10.1111/j.1600-0587.2013.07872.x, 2013.	This model uses four Millenium Ecosystem Assesssment scenarios and their related IPCC scenarios to evaluate the effect of climate change and land conversion by 2100 on 8750 birds species distribution. "We integrated the exposure of species to climate and land-use change through the combined effects of these drivers on global land cover and explored the resulting reductions in range size and possible extinctions within the world's 8,750 terrestrial bird species".<br>MA scenario evaluations are based on IMAGE 2.2 model	a. biodiversity model	1985-2050, 1985-2100	1km2	Global
BIOMOD2 (BIOdiversity MODelling)	Thuiller 2004.<br>Thuiller, W.: Patterns and uncertainties of species' range shifts under climate change, Glob. Change Biol., 10, 2020–2027, https://doi.org/10.1111/j.1365-2486.2004.00859.x, 2004.<br><br>Thuiller et al. 2009<br>Thuiller, W., Lafourcade, B., Engler, R., and Araújo, M. B.: BIOMOD – a platform for ensemble forecasting of species distributions, Ecography, 32, 369–373, https://doi.org/10.1111/j.1600-0587.2008.05742.x, 2009.<br><br>Thuiller et al. 2011.<br>Thuiller, W., Lavergne, S., Roquet, C., Boulangeat, I., Lafourcade, B., and Araujo, M. B.: Consequences of climate change on the tree of life in Europe, Nature, 470, 531–534, https://doi.org/10.1038/nature09705, 2011.	Platform for ensemble forecasting of species distribution, able to fit and compare different models and incorporates several features for testing models and for examining species-environment relationships. The output is a projection of potential distribution of species into future environmental conditions	a. biodiversity model	Depends on the time lapse for predicted future environmental conditions	Flexible	Flexible
cSAR (Countryside Species Area Relationship) - iDiv	Pereira, H.M., Ziv, G.U.Y. and Miranda, M., 2014. Countryside species–area relationship as a valid alternative to the matrix-calibrated species–area model. Conservation Biology, 28(3), p.874.<br>Pereira, H. M., and G. C. Daily. 2006. Modeling biodiversity dynamics in countryside landscapes. Ecology 87:1877–1885.	Species area relationship model considering habitat affinity of functional groups into the richness equation. This model predicts how many species are likely to be lost by functional group.	a. biodiversity model	Depends on habitat conversion time scale	Flexible	Flexible

cSAR-IIASA-ETH	Chaudhary, A., Verones, F., de Baan, L. and Hellweg, S.: Quantifying Land Use Impacts on Biodiversity: Combining Species–Area Models and Vulnerability Indicators, Environ. Sci. Technol., 49(16), 9987–9995, doi:10.1021/acs.est.5b02507, 2015.	Uses cSAR model and provides an updated impact assessment approach and equations for the characterization factors for regional and global biodiversity loss which give an estimate of regional species loss per unit of land use and land use change	a. biodiversity model	Depends on land use change time scale	Flexible	Flexible
BILBI (Biogeographic modelling Infrastructure for Large-scale Biodiveristy Indicators)	Hoskins, A.J., Harwood, T.D., Ware, C., Williams, K.J., Perry, J.J., Ota, N., Croft, J.R., Yeates, D.K., Jetz, W., Golebiewski, M. and Purvis, A., 2020. BILBI: Supporting global biodiversity assessment through high-resolution macroecological modelling. Environmental Modelling & Software, p.104806.<br><br>Di Marco M, Harwood TD, Hoskins AJ, Ware C, Hill SLL, Ferrier S (2019) Projecting impacts of global climate and land-use scenarios on plant biodiversity using compositional-turnover modelling. Global Change Biology 25: 2763-2778.	Uses species-area relationship and generalized dissimilarity modeling to model spatial and temporal turnover of species. Space-for-time substitutions can be used to estimate the impacts of land use change and climate change on beta diversity	a. biodiversity model	Flexible	30-arcsecond (~1km)	Global terrestrial
PREDICTS (Projecting Responses of Ecological Diversity In Changing Terrestrial Systems)	Newbold, T., L. N. Hudson, S. L. L. Hill, S. Contu, I. Lysenko, R. A. Senior, L. Borger, D. J. Bennett, A. Choimes, B. Collen, J. Day, A. De Palma, S. Díaz, S. Echeverria-Londono, M. J. Edgar, A. Feldman, M. Garon, M. L. K. Harrison, T. Alhusseini, D. J. Ingram, Y. Itescu, J. Kattge, V. Kemp, L. Kirkpatrick, M. Kleyer, D. L. P. Correia, C. D. Martin, S. Meiri, M. Novosolov, Y. Pan, H. R. P. Phillips, D. W. Purves, A. Robinson, J. Simpson, S. L. Tuck, E. Weiher, H. J. White, R. M. Ewers, G. M. Mace, J. P. W. Scharlemann, and A. Purvis. 2015. Global effects of land use on local terrestrial biodiversity. Nature 520:45-50.	Statisical models that predict biodiversity (i.e., richness, abundance, CWM size) as a function of land use and infrastructure, based on spatial observations of biodiversity. Each study site was scored for six anthropogenic pressures: use history, human population density, proximity to roads, and accessibility from the nearest large town. Random effects accounted for study-level differences in response variables and sampling methods, and for the within-study spatial arrangement of sites.	a. biodiversity model	these are spatial, rather than temporal, comparisons	Sites varied in the maximum linear extent sampled (median 106 m; inter- quartile range 50 m to 354 m)	global terrestrial
GLOBIO (BLObal BIOdiversity) - Aquatic	Janse, J. H., Kuiper, J. J., Weijters, M. J., Westerbeek, E.P., Jeuken, M. H. J. L., Bakkenes, M., Alkemade, R.,Mooij, W. M. and Verhoeven, J. T. A.: GLOBIO-Aquatic, a global model of human impact on the biodiversity of inland aquatic ecosystems, Environ. Sci. Policy, 48, 99–14, https://doi.org/10.1016/j.envsci.2014.12.007, 2015.<br>Janse, J. H., Bakkenes, M., and Meijer, J.: Globio-Aquatic, Technical model description v. 1.3, PBL publication 2829, The Hague,PBL Netherlands Environmental Assessment Agency, 2016.	Quantifies the impacts of multiple anthropogenic pressures on freshwater biodiversity and its ecosystem services. The drivers included are land use, eutrophication, climate change and hydrological disturbance. The model uses correlative relationships between anthropogenic drivers and biodiversity and ecosystem services of rivers, lakes and wetlands. The model produces biodiversity intactness indicator – Mean Species Abundance (MSA) – of lakes, rivers and wetlands as well as the probability of harmful algal blooms	a. biodiversity model	Could be applied at different temporal scales	0.5°	global
GLOBIO - Terrestrial	Schipper, A.M., Bakkenes, M., Meijer, J.R., Alkemade, R., Huijbregts, M.J.: The GLOBIO model. A technical description of<br>version 3.5. PBL publication 2369, The Hague, PBL Netherlands Environmental Assessment Agency, 2016.<br>Alkemade, R., van Oorschot, M., Miles, L., Nellemann, C., Bakkenes, M., and ten Brink, B.: GLOBIO3: A Framework to Investigate Options for Reducing Global Terrestrial Biodiversity Loss, Ecosystems, 12, 374–390, https://doi.org/10.1007/s10021- 009-9229-5, 2009.	Projecting terrestrial biodiversity intactness with GLOBIO 4. Originally developed to quantify the impacts of infrastructure on biodiversity intactness, later extended to also include the impacts of climate change, land use and atmospheric nitrogen deposition (and impacts of hunting in tropical regions). Quantifies biodiversity using the Mean Species Abundance (MSA).<br>Impact relationships for terrestrial plants and warm-blooded vertebrates (birds and mammals)<br>GLOBIO is built on a set of equations linking environmental drivers (modeled by IMAGE) and biodiversity impact (cause-effect relationships)	a. biodiversity model	2000, 2005, 2050	~300m	Global excluding Antarctica and Greenland

DBEM (Dynamic Bioclimate Envelope Model)	Cheung, W. W. L., Dunne, J., Sarmiento, J. L. and Pauly, D.(2011) Integrating ecophysiology and plankton dynamics into projected maximum fisheries catch potential under climate change in the Northeast Atlantic, ICES J. Mar. Sci., 68(6), 1008–1018.	Defines a bioclimatic envelope for each species, and simulates changes in abundance and carrying capacity under a varying environment. Bbrings in niches, produciton, dispersal, population growth and some habitat fields but does not include trophic interactions (biodiversity derived by layering up multiple species)	a. biodiversity model	Annual	½ × ½ degree (implicit depth via niche definitions)	Global
Madingley	Harfoot et al., 2014, PLoS Biology; Bartlett et al., 2016, Proc Roy Soc; Newbold et al., 2020, Science Advances	Focuses on both biodiversity and ecosystem functioning. Model describes organisms according to a set of functional traits. Ecological processes are described at the level of individual organisms and they are applied to groups of individuals, cohorts, the fundamental agents in the model. Higher level properties from the model emerge from the interactions between cohorts, as mediated by the environment in a given location.	b. ecosystem function model	Monthly	Variable but typically 0.25 x 0.25 - 2 x 2 dd	Global (land and sea)
LPJ-GUESS (Lund-Potsdam-Jena General Ecosystem Simulator)	Smith, B., Prentice, I.C. & Sykes, M.T. 2001. Representation of vegetation dynamics in the modelling of terrestrial ecosystems: comparing two contrasting approaches within European climate space. Global Ecology & Biogeography 10: 621-637. Other relevant sources with different updates and components can be found at http://web.nateko.lu.se/lpj-guess/resources.html	LPJ-GUESS is a process-based dynamic vegetation-terrestrial ecosystem model designed for regional or global studies. Outputs include vegetation composition and cover in terms of major species or plant functional types (PFTs), biomass and soil organic matter carbon pools, leaf area index (LAI), net primary production (NPP), net ecosystem carbon balance, carbon emissions from wildfires, biogenic volatile organic compounds (BVOCs), evapotranspiration, runoff, and nitrogen pools and fluxes. The latest version (4.0) includes functionality for “managed land”. Specific versions of the model include other outputs such as methane emissions, and permafrost.	b. ecosystem function model	Daily, monthly, yearly	Flexible; Patches (0.1 hectare); Individual tree	Global (and regional)
LPJ (Lund-Potsdam-Jena)	Sitch et al. 2003. Global Change Biology, Evaluation of ecosystem dynamics, plant geography and terrestrial carbon cycling in the LPJ dynamic global vegetation model.	LPJ is a dynamic global vegetation model. It represents global plant diversity as a set of plant functional types and simulates their distribution and biogeochemical cycles across the world.	b. ecosystem function model	Daily	1x1	Global
CABLE (Community Atmosphere Biosphere Land Exchange)	Wang et al. 2011, Journal of Geophysical research. Diagnosing errors in a land surface model (CABLE) in the time and frequency domains. 116, G01034, doi:10.1029/2010JG001385.<br>Haverd, V., Smith, B., Nieradzik, L., Briggs, P. R., Woodgate, W., Trudinger, C. M., Canadell, J. G., and Cuntz, M.: A new version of the CABLE land surface model (Subversion revision r4601) incorporating land use and land cover change, woody vegetation demography, and a novel optimisation-based approach to plant coordination of photosynthesis, Geosci. Model Dev., 11, 2995–3026, https://doi.org/10.5194/gmd-11-2995-2018, 2018.	CABLE is the land model of the Earth system model ACCESS (Australian Community Climate and Earth System Simulator). It simulates land surface energy blance, water budget, and carbon cycle, and vegetation dynamics.	b. ecosystem function model	hourly/half hourly	(2x2.5 ?)	Global
Macroecological model	Jennings, S. and Collingridge, K. (2015) Predicting consumer biomass, size-structure, production, catch potential, responses to fishing and associated uncertainties in the world’s marine ecosystems., PLoS One, 10(7), e0133794	A static model, predicts mean size composition and abundance of marine animals (using size spectra) based on minimal inputs (e.g. available primary production) nd metabolic scaling theory	b. ecosystem function model	Static equilibrium prediction	½ × ½ degree (2D as surface integrated)	Global

POEM	Watson, J. R., Stock, C. A. and Sarmiento, J. L. (2015) Exploring the role of movement in determining the global distribution of marine biomass using a coupled hydrodynamic - Size-based ecosystem model, Prog. Oceanogr., 138, 521–532	Size-based model - using empirical allometric relationships to govern model ecological interactions.	b. ecosystem function model	Daily	1 x 1 degree (surface ocean only)	Global
SEAPODYM	Lehodey, P., Murtugudde, R. and Senina, I. (2010) Bridging the gap from ocean models to population dynamics of large marine predators: A model of mid-trophic functional groups, Prog. Oceanogr., 84(1–2), 69–84	Eulerian model linking lower and mid-trophic level functional groups with IBMs for populations dynamics of target species (e.g. tunas)	b. ecosystem function model	daily or quarterly	1/12 x 1/12 or 1-2 x1-2 degree resolutions possible. 3 vertical layers: epi- and mesopelagic ocean.	Regional or global
GLOBIO Ecosystem Services	Schulp, C. J. E., Alkemade, R., Klein Goldewijk, K., and Petz, K.: Mapping ecosystem functions and services in Eastern Europe using global-scale data sets, Int. J. Biodivers. Sci. Ecosyst. Serv. Manag., 8, 156–168,https://doi.org/10.1080/21513732.2011.645880, 2012.	Methodology to map, quantify and simulate Ecosisten functions (ESFs) and Ecosystem services (ESSs). Includes the extent of the human use of ecosystems and it is related to the link between ecosystem functioning and human wellbeing. Estimation of provisioning, regulating and cultural services, focusig on services that depend on the landscape structure They evaluate the correlation between ESF availability and ESS supply (only for cultural services high ESF areas overlap with high ESS supply)	c. ecosystem services model		0.5 x 0.5 °	Eastern Europe
InVEST Carbon (Integrated Valuation of Ecosystem Services and Tradeoffs)	http://releases.naturalcapitalproject.org/invest-userguide/latest/carbonstorage.html#	Model estimates the current amount of carbon stored in a landscape and values the amount of sequestered carbon over time	c. ecosystem services model	Flexible	Flexible	Flexible
InVEST Fisheries (Integrated Valuation of Ecosystem Services and Tradeoffs)	http://releases.naturalcapitalproject.org/invest-userguide/latest/fisheries.html	Model produces estimates of harvest volume and economic value of single-species fisheries	c. ecosystem services model	Flexible	Flexible	Flexible
InVEST Pollination (Integrated Valuation of Ecosystem Services and Tradeoffs)	http://releases.naturalcapitalproject.org/invest-userguide/latest/croppollination.html	Model estimates an index of abundance of bees in each cell of the study site and the contribution of these bees to agricultural production. Can be used to understand how changes in land use and agricultural management can impact crop pollination and yield.	c. ecosystem services model	Flexible	Flexible	Flexible
GLOSP (GLObal Soil Protection)	Guerra, C. A., Maes, J., Geijzendorffer, I., and Metzger, M. J.: An assessment of soil erosion prevention by vegetation in Mediterranean Europe: Current trends of ecosystem service provision, Ecol. Indic., 60, 213–222, https://doi.org/10.1016/j.ecolind.2015.06.043, 2016.	Model of soil erosion prevention in Mediterranean Europe between 2001 and 2013, based on eight process-based indicators	c. ecosystem services model	2001,2005,2009 ,2013	5x5Km2	European Mediterranean
SS-DBEM (size-spectrum and DBEM model)	Fernandes, J. A., Cheung, W. W. L., Jennings, S., Butenschön, M., De Mora, L., Frölicher, T. L., Barange, M. and Grant, A. (2013) Modelling the effects of climate change on the distribution and production of marine fishes: Accounting for trophic interactions in a dynamic bioclimate envelope model, Glob. Chang. Biol., 19(8), 2596–2607	Combines the DBEM and the "Macroecological model" size spectrum model - projects changes in species distribution, abundance and body size, and includes populations dynamics, dispersal, competition and predation.	c. ecosystem services model	Annual	½ × ½ degree (implicit depth via niche definitions)	Global
EwE (Ecopath with Ecosim)	Christensen, V. and Walters, C. J. (2004) Ecopath with Ecosim: methods, capabilities and limitations, Ecol. Modell., 172(2–4), 109–139	A mass-balance foodweb (trophic) model that accounts for the flow of biomass between trophic groups (species or functional gruops, as defined by the modeller).	c. ecosystem services model	~Monthly	Non spatial (can be converted to 2D spatial Ecospace, but not done in many models)	Regional

EcoOcean	Christensen, V., Coll, M., Buszowski, J., Cheung, W. W. L., Frölicher, T., Steenbeek, J., Stock, C. A., Watson, R. A. and Walters, C. J. (2015) The global ocean is an ecosystem: simulating marine life and fisheries, Glob. Ecol. Biogeogr., 24(5), 507– 517	A gridded global food web model based on the EwE framework, designed to evaluate the impact of climate change and human pressure on marine ecosystems.	c. ecosystem services model	~Monthly (can be Annual)	½ × ½ degree (implicat depth reperesenttion via alllowed feeding interactions)	Global
DPBM (Dynamic Pelagic-Benthic Model)	Blanchard, J. L., Jennings, S., Holmes, R., Harle, J., Merino, G., Allen, J. I., Holt, J., Dulvy, N. K. and Barange, M. (2012) Potential consequences of climate change for primary production and fish production in large marine ecosystems, Philos. Trans. R. Soc. London B Biol. Sci., 367(1605), 2979–2989	A functional trait-based size spectrum model (joins a classic pelagic predator size spectra model with a benthic detritivore size spectrum) and macroecological physiological allocation of resources within size classes of the trait groups; can include unstructured resources and groups (e.g. herbivores) that do not feed according to size.	c. ecosystem services model	Daily or weekly	Flexible - e.g. ½ × ½ degree (2D following sea surface and seabed)	Global or regional
BOATS (BiOeconomic mArine Trophic Sizespectrum model)	Carozza, D. A., Bianchi, D. and Galbraith, E. D.(2016) The ecological module of BOATS-1.0: a bioenergetically-constrained model of marine upper trophic levels suitable for studies of fisheries and ocean biogeochemistry, Geosci. Model Dev., 9, 1545–1565	Size-based model with metabolic constraints - projects production of fish, resolved across multiple size spectra, with coupled econimic model (for fleet dynamics)	c. ecosystem services model	Monthly	Flexible - e.g. ½ × ½ or 1 x 1 degree (2D as surface integrated)	Global (typically)
Atlantis	Fulton, E. a, Link, J. S., Kaplan, I. C., Savina-Rolland, M., Johnson, P., Ainsworth, C., Horne, P., Gorton, R., Gamble, R. J., Smith, A. D. M. and Smith, D. C. (2011) Lessons in modelling and management of marine ecosystems: the Atlantis experience, Fish Fish., 12(2), 171–188; Fulton, E.A. and Gorton, R., 2014. Adaptive Futures for SE Australian Fisheries & Aquaculture: Climate Adaptation Simulations. CSIRO, Australia. pp 309	Whole of ecosystem model - mechanistically follows major biogeochemical and ecological processes: modular implementation (multiple options per process). Includes age structure, full life history closure, gape-limited predation, habitats depenency movement and a range of human use (especially fisheries) representatoins	c. ecosystem services model	Flexible, typically 6-24 hourly	Variable (based on polygons) - 3D with polygon size and shape matched to biophysical features;.Vertical profile resolved using "slab" layers (with finer layers at the surface and thicker at depth).	Regional
OSMOSE (Object-oriented Simulator of Marine ecOSystems Exploitation)	Travers, M., Shin, Y. J., Jennings, S., Machu, E., Huggett, J. A., Field, J. G. and Cury, P. M. (2009) Two-way coupling versus one-way forcing of plankton and fish models to predict ecosystem changes in the Benguela, Ecol. Modell., 220(21), 3089–3099	Individual-based multi-species fish model coupled to bioogeochemcial plankton model	c. ecosystem services model	weekly	1/6 × 1/6 degree (2D as surface integrated)	Regional
APECOSM	Maury, O. (2010) An overview of APECOSM, a spatialized mass balanced "Apex Predators ECOSystem Model" to study physiologically structured tuna population dynamics in their ecosystem, Prog. Oceanogr., 84(1–2), 113–117	Eulerian model of size structured marine populations and communities (size-spectrum model) coupled with IBM approaches to represent environmentally driven individual bioenergetics, trophic interactions and behaviours (like schooling); with tuna as top predstor focus	c. ecosystem services model	12-24 hour	Can be run on any spatial grid from regional to global scale. Implicit depth representation via explicitly parameterized movement dynamics	Regional or global

Model Details

Model Name	Variable Name	Variable Description	Input/Output	Variable Class	Biodiversity Dependent Variable?
AIM-biodiversity	Climate variables	monthly minimum temperature, maximum temperature, and precipitation	input	pressure	No
AIM-biodiversity	Land cover	Regional aggregated land use using Asia-Pacific Integrated Model/Computable General Equilibrium Model (AIM/CGE). Classes include cropland, pasture, forest, other natural land, settled land.	input	pressure	No
AIM-biodiversity	Species occurrence	Species occurrence records from GBIF	input	biodiversity	NA
AIM-biodiversity	Dispersal ability	Species level dispersal ability based on life history traits	input	biodiversity	NA
AIM-biodiversity	Change in suitable habitat	Area lost or gained of suitable habitat due to land use change and climate change	output	pressure	No
InSIGHTS	Land cover	Globcover v. 2.1. 300m map with 63 classes based on standard UN Land Cover Classificaction System (LCCS)	input	pressure	No
InSIGHTS	Elevation	Remapled to 300m the Shuttle Radar Topography Mission elevation of aprox 90m resolution	input	EF	No
InSIGHTS	Hydrological features	Merged from 300m wide buffer arround 210 class of Globcover water bodies and Vmap0 linear permanent water map for linear water bodies (converted to 300m)	input	EF	No
InSIGHTS	Habitat preferences		input	habitat	NA
InSIGHTS	Species richness		output	biodiversity	NA
MOL	CO_2 emissions	global Gt of CO_2 emissions per year	input	pressure	No
MOL	ΔT	change in average annual temperature compared to 1970 in °C	input	pressure	No
MOL	Land-cover change (Clim)	percentage of global terrestrial land transformed by climate change	input	pressure	No
MOL	Land-cover change (Hab)	percentage of global terrestrial land transformed by human land-use change	input	pressure	No

MOL	Species distribution range		input	biodiversity	NA
MOL	Projected distribution range		output	biodiversity	NA
BIOMOD2	Species distribution	Presence/abscense (pseudo-absences)	input	biodiversity	NA
BIOMOD2	Environmental data - current	climate, land use change	input	pressure	No
BIOMOD2	Environmental data - future scenarios	climate, land use change	input	pressure	No
BIOMOD2	Species potential distribution	Future projection	output	biodiversity	NA
cSAR	Number of species lost by group		output	biodiversity	NA
cSAR	Afinity of functional groups to habitat	Proportion of area of habitat that is usable by a functional group	input	habitat	NA
cSAR	Area of habitat in the landscape		input	pressure	Yes
cSAR	Area of habitat after conversion		input	pressure	No
cSAR	Number of species groups in area of interest		input	biodiversity	NA
cSAR	Dispersal variance		input	biodiversity	NA
cSAR	Sensitivity of the taxon to the transformed habitat		input	biodiversity	NA
cSAR	z	Constant from classic SAR model for the area of interest	input	NA	NA
cSAR-IIASA-ETH	Local characterization factor		output	biodiversity	NA
cSAR-IIASA-ETH	Species richness per ecoregion		input	biodiversity	NA
cSAR-IIASA-ETH	Original natural habitat area		input	pressure	Yes
cSAR-IIASA-ETH	Remaining natural habitat area		input	pressure	Yes
cSAR-IIASA-ETH	Area per land use type		input	pressure	No

cSAR-IIASA-ETH	z	Constant from classic SAR model for the area of interest	input	NA	NA
cSAR-IIASA-ETH	Regeneration time (treg)		input	EF	Yes
cSAR-IIASA-ETH	Vulnerability score (VS)		output	biodiversity	NA
BILBI	Species occurrence records	Species occurrence records from GBIF and Map of Life	input	biodiversity	NA
BILBI	Soil properties	bare ground, bulk density, clay, pH, silt	input	EF	No
BILBI	Terrain variables	topographic roughness index, topographic wetness index	input	EF	No
BILBI	Climate variables	annual precipitation, annual minimum temperature, annual maximum temperature, maximum monthly diurnal temperature range, annual actual evapotranspiration, potential evaporation of the driest month, maximum and minimum monthly water deficit	input	pressure	No
BILBI	Compositional turnover	Compositional turnover across space or time	output	biodiversity	NA
Madingley	Land use	Time series of fractional coverage of grid cells by land use, current in three classes: primary, secondary and impacted	input	pressure	No
Madingley	Climate variables	Monthly time series of climatic variables: temperature (air and ocea), diurnal temperature range, precipitation, frost days	input	pressure	No
Madingley	Biomass (per cohort or functional group within or across spatial cells)		output	biodiversity	NA
Madingley	Abundance (per cohort or functional group within or across spatial cells)		output	biodiversity	NA
Madingley	Size structure		output	biodiversity	NA
Madingley	Trophic structure		output	biodiversity	NA
Madingley	Functional richness		output	biodiversity	NA

Madingley	Functional intactness		output	biodiversity	NA
Madingley	Functional Beta diversity		output	biodiversity	NA
Madingley	Ecological process rates	e.g. Feeding interactions (herbivory, carnivory, insectivory etc)	output	EF	Yes
Madingley	Ecological process rates	e.g. Metabolism and biomass turnvover rates	output	EF	Yes
Madingley	Ecological resilience/stability		output	EF	Yes
Globio-Terrestrial	climate change		input	pressure	No
Globio-Terrestrial	land use		input	pressure	No
Globio-Terrestrial	fragmentation		input	pressure	No
Globio-Terrestrial	road disturbance		input	pressure	No
Globio-Terrestrial	nitrogen deposition		input	pressure	Yes
Globio-Terrestrial	hunting		input	pressure	No
Globio-Terrestrial	Mean species abundance		output	biodiversity	NA
Globio-Terrestrial	Species abundance (undisturbed)		input	biodiversity	NA
Globio-Terrestrial	Species abundance (impacted)		input	biodiversity	NA
GLOBIO Ecosystem Services	Food crop yield	Mg/km2 per growth cycle in fresh weight	output	ES	Yes
GLOBIO Ecosystem Services	Wild food	Amount of wild food (game, fish, berries and mushrooms (kg/km2)) accessible to people within the maximum amount of time that people spend for collecting wild food (between 0.5 to 2 hours depending of the food)	output	ES	Yes
GLOBIO Ecosystem Services	Carbon sequestration	Net ecosystem productivity (Mg C/km2 per year)	output	ES	Yes
GLOBIO Ecosystem Services	Protection against erosion		output	ES	Yes
GLOBIO Ecosystem Services	Flood protection		output	ES	Yes

GLOBIO Ecosystem Services	Pollination		output	ES	Yes
GLOBIO Ecosystem Services	Air quality		output	ES	Yes
GLOBIO Ecosystem Services	Tourism and recreation		output	ES	Yes
GLOBIO Ecosystem Services	Land cover	Global land cover map at 250m res	input	pressure	No
GLOBIO Ecosystem Services	Elevation	global DEM at 1km res	input	EF	No
GLOBIO Ecosystem Services	Precipitation sum	Annual precipitation sum, 0.5° res	input	pressure	No
GLOBIO Ecosystem Services	Precipitation surplus	Annual precipitation sum minus annual evapotranspiration, 0.5° res	input	pressure	No
GLOBIO Ecosystem Services	Precipitation distribution	L% of annual precipitation per month, 0.5° res	input	pressure	No
GLOBIO Ecosystem Services	Wet day frequency	Number of rain days per year	input	pressure	No
GLOBIO Ecosystem Services	Temperature	Annual mean temperature, 0.5°res	input	pressure	No
GLOBIO Ecosystem Services	Rivers	Location and hierrarchy of rivers	input	EF	No
GLOBIO Ecosystem Services	Coasts	Land-sea boundary	input	EF	No
GLOBIO Ecosystem Services	Soil characteristics	Clay, silt and sand content; rooting depth, bulk density, from the Harmonized world soil database version 1.0, 30"res	input	EF	No

GLOBIO Ecosystem Services	Population density	Number of peiple per pixel at 1 ar second res	input	pressure	No
GLOBIO Ecosystem Services	Crop fraction	For each crop included in IMAGE, precentage of agricultural land in each IMAGE grid cell covered by this particular crop. Crops sincluded: cereals, rice, maize, pulses, roots and tubers, oil crops	input	ES	No
GLOBIO Ecosystem Services	GDP	Gross domestic product per country and NUTS2 region	input	economic	No
GLOBIO Ecosystem Services	Roads	Location and type of roads from GRIP	input	pressure	No
GLOBIO Ecosystem Services	Management factor	Management intensity (IMAGE); crop specific and region specific	input	pressure	No
GLOBIO-Aquatic	Map of surface water bodies	Location and types of aquatic ecosystems (lakes, rivers, wetlands)	input	EF	No
GLOBIO-Aquatic	Lake depths	Lake depths in m	input	EF	No
GLOBIO-Aquatic	Digital water network	To calculate movement of water across globe	input	EF	No
GLOBIO-Aquatic	Land use	Laud use and land cover	input	pressure	No
GLOBIO-Aquatic	Map of major river dams		input	pressure	No
GLOBIO-Aquatic	Water discharge	used to calculate river flow deviation, monthly	input	pressure	No
GLOBIO-Aquatic	Water discharge in natural sitaution	used to calculate river flow deviation, monthly	input	pressure	No
GLOBIO-Aquatic	Phosphorus	Phosphorus concentration in surface water (g P m-3), year-averages	input	pressure	No
GLOBIO-Aquatic	Nitrogen	Nitrogen concentration in surface water (g N m-3), year-averages	input	pressure	No
GLOBIO-Aquatic	Water temperature	degrees C, monthly	input	pressure	No
GLOBIO-Aquatic	Mean species abundance	measure of biodiversity intactness	output	biodiversity	NA
GLOBIO-Aquatic	Concentration of harmful agae in lakes	g/m3	output	EF	Yes
InVEST-pollination	Land Cover	Raster of LULC for each pixel	input	pressure	No

InVEST-pollination	Nesting availability index by substrate type	Relative index of the availability of the given nesting type within each LULC type, on a floating point scale of 0-1	input	pressure	Yes
InVEST-pollination	Floral resources by season	Relative abundance of flowers in each LULC class for a given season	input	pressure	Yes
InVEST-pollination	Nesting suitability index by species/guild type	Index of nesting suitability for each species or guild	input	EF	Yes
InVEST-pollination	Foraging activity by season	Pollinator activity index by floral season (i.e. flight season)	input	EF	Yes
InVEST-pollination	Distance traveled by each species	Average distance each species or guild travels to forage on flowers, in meters. Can be determined by typical foraging distance of a bee species based on allometric relationship.	input	EF	Yes
InVEST-pollination	Bee relative abundance	weighted relative abundance of each species to total pollinator abundance.	input	biodiversity	NA
InVEST-pollination	Farm vector	Farm vector shapefile with the follwoing information about the farm: crop type, half saturation coefficient (value of pollinator abundance at which 50% of pollinator-dependent crop obtained), season crop is pollinated, floral resources available for the given season, substrate suitability for farm for given substrate, proportion of crop dependent on pollinators, and proportion of pollination required on teh farm provided by managed pollinators.	input	EF	Yes
InVEST-pollination	Pollinator abundance	average pollinator abundance on farm for the active season, and per pixel abundance of each pollinator species in the season	output	biodiversity	NA
InVEST-pollination	Pollinator supply	per pixel index of pollinator species that could be on a pixel given its abundance factor from table, habitat suitability for that species, and multiplied by available flora resources that pollinator could fly to from that pixel	output	biodiversity	NA

InVEST-pollination	Total pollinator yield	per pixel total pollinator yield	output	ES	Yes
InVEST-pollination	Wild pollinator yield	per pixel pollinator yield for wild pollinators only	output	ES	Yes
InVEST-carbon	Land Cover	Raster of LULC for each pixel. Current anf future scenarios	input	pressure	No
InVEST-carbon	Carbon pools	Table of LULC classes with data on C stored in each of the 4 fundamental pools for each class.	input	EF	Yes
InVEST-carbon	Price/metric ton of C	Price given in currency per metric ton of elemental carbon	input	economic	No
InVEST-carbon	Market discount price C	An integer percentage value which reflects society’s preference for immediate benefits over future benefits	input	economic	No
InVEST-carbon	Annual rate of change in price of C	An integer percentage value which adjusts the value of sequestered carbon as the impact of emissions on expected climate change-related damages changes over time	input	economic	No
InVEST-carbon	C storage	C stored in Mg in each pixel for all LULC scenarios. Available as total C and as difference between scenarios. Also available in C storage in each of the 4 C pools.	output	EF	Yes
InVEST-carbon	Economic value of C sequestered	Economic value of C sequestered between current and future landscape dates. units are in currency per pixel	output	ES	No
InVEST-fisheries	Basic model parameters	Age or stage structure, sex specific, harvest by individuals or weight, #time steps	input	NA	No
InVEST-fisheries	Population parameters	Age/stage classes; survival and natural mortality rates	input	biodiversity	NA
InVEST-fisheries	Subregion attributes	exploitation fraction by subregion; larval dispersal by subregion	input	biodiversity	NA
InVEST-fisheries	Class-specific attributes	Vulnerability to harvest by age class; fraction of age class that is mature (contributes to spawning stock); duration of stage for stage-class models; average biomass of each age/stage; fecundity	input	biodiversity	NA

InVEST-fisheries	Recruitment parameters	Initial number of recruits; recruitment function type; spawners by individuals or weight; recurring number of recruits	input	biodiversity	NA
InVEST-fisheries	Migration parameters	source/sink migration matrix by age/stage class	input	biodiversity	NA
InVEST-fisheries	Valuation parameters	fraction of harvest after processing; unit price	input	economic	No
InVEST-fisheries	Habitat dependency parameters	Habitat dependencies (0-1) for each age/stage for each habitat type	input	habitat	NA
InVEST-fisheries	Habitat area change	percent change in each habitat type by subregion	input	pressure	No
InVEST-fisheries	Fish harvest	cumulative harvest (weight or individuals) across area of interest per time step; fina harvest by subregion	output	ES	Yes
InVEST-fisheries	Fish harvest value	valuation of each subregion harvest	output	ES	Yes
GLOSP (GLObal Soil Protection)	Local and regional climate, topography and soil characteristics	Rainfall erosivity factor, soil erodibility, topography, vegetation cover	input	pressure	No
GLOSP (GLObal Soil Protection)	Structural impact	Total soil erosion impact when no ES is provided	output	ES	No
GLOSP (GLObal Soil Protection)	Ecosystem service mitigated impact	Total of the remaining soil erosion after the ecosystem service provision	output	ES	Yes
GLOSP (GLObal Soil Protection)	Actual ecosystem service provision	Total amount of soil erosion prevention provided	output	ES	Yes
GLOSP (GLObal Soil Protection)	Ecosystem service provision capacity	Average fraction of the structural impact that is mitigated by soil erosion prevention	output	ES	Yes
GLOSP (GLObal Soil Protection)	Variation in structural impact	Percentage of variation in the total amount of structural impact	output	ES	No
GLOSP (GLObal Soil Protection)	Rate of effective ecosystem service provision	Percentage of variation in the total amount of actual ecosystem service provision corrected by the structural impact fluctuations	output	ES	Yes
GLOSP (GLObal Soil Protection)	Variation in ecosystem service provision capacity	Percentage of variation in the total amount of ecosystem service provision capacity considering the previous reference date	output	ES	Yes

GLOSP (GLObal Soil Protection)	Variation in ecosystem mitigated impact	Percentage of variation in the total amount of ecosystem service mitigated impact considering the previous reference date	output	ES	Yes
DBEM (Dynamic Bioclimate Envelope Model)	Biomass (per species per spatial cell)		output	EF	Yes
DBEM (Dynamic Bioclimate Envelope Model)	Catch (per species per spatial cell)		output	ES	Yes
DBEM (Dynamic Bioclimate Envelope Model)	Primary production		input	EF	Yes
DBEM (Dynamic Bioclimate Envelope Model)	Environmental conditions (e.g. temperature)		input	pressure	No
SS-DBEM (size-spectrum and DBEM model)	Biomass (per species per spatial cell)		output	EF	Yes
SS-DBEM (size-spectrum and DBEM model)	Catch (per species per spatial cell)		output	ES	Yes
SS-DBEM (size-spectrum and DBEM model)	Primary production		input	EF	Yes
SS-DBEM (size-spectrum and DBEM model)	Environmental conditions (e.g. temperature)		input	pressure	No
EwE (Ecopath with Ecosim)	Biomass (per species/functional group or stanza where age structure included)		output	EF	Yes
EwE (Ecopath with Ecosim)	Catch (per species/functional group or stanza where age structure included)		output	ES	Yes

EwE (Ecopath with Ecosim)	Estimates of predation (per species/functional group)		output	EF	Yes
EwE (Ecopath with Ecosim)	Estimates of fishing mortality (per species/functional group)		output	pressure	No
EwE (Ecopath with Ecosim)	Estimates of diets/consumption (per species/functional group)		output	EF	Yes
EwE (Ecopath with Ecosim)	Estimates of simple indices of biodiversity (Kempton's Q)		output	biodiversity	NA
EwE (Ecopath with Ecosim)	Primary production		input	EF	Yes
EwE (Ecopath with Ecosim)	Environmental conditions (e.g. temperature)		input	pressure	No
EcoOcean	Biomass (per species or stanza where age structure included, per spatial cell)		output	EF	Yes
EcoOcean	Catch (per species or stanza where age structure included, per spatial cell)		output	ES	Yes
EcoOcean	Primary production		input	EF	Yes
EcoOcean	Environmental conditions (e.g. temperature)		input	pressure	No
Macroecological model	Biomass (per size class)		output	EF	Yes
Macroecological model	Primary production		input	EF	Yes

Macroecological model	Environmental conditions (e.g. temperature)		input	pressure	No
DPBM (Dynamic Pelagic-Benthic Model)	Biomass (per size classand trait group per spatial cell)		output	EF	Yes
DPBM (Dynamic Pelagic-Benthic Model)	Catch (per size classand trait group per spatial cell)		output	ES	Yes
DPBM (Dynamic Pelagic-Benthic Model)	Biodiversity proxy (slope of size spectrum)		output	biodiversity	NA
DPBM (Dynamic Pelagic-Benthic Model)	Primary production		input	EF	Yes
DPBM (Dynamic Pelagic-Benthic Model)	Environmental conditions (e.g. temperature)		input	pressure	No
BOATS (BiOeconomic mArine Trophic Sizespectrum model)	Biomass (per size class per spatial cell)		output	EF	Yes
BOATS (BiOeconomic mArine Trophic Sizespectrum model)	Catch (per size class per spatial cell)		output	ES	Yes
BOATS (BiOeconomic mArine Trophic Sizespectrum model)	Primary production		input	EF	Yes
BOATS (BiOeconomic mArine Trophic Sizespectrum model)	Environmental conditions (e.g. temperature)		input	pressure	No
POEM	Biomass (per size class per spatial cell)		output	EF	Yes

POEM	Catch (per size class per spatial cell)		output	ES	Yes
POEM	Primary production		input	EF	Yes
POEM	Environmental conditions (e.g. temperature)		input	Pressure	No
Atlantis	Biomass (per size and age class per species per spatial cell; including primary producers and habitat forming groups)		output	EF	Yes
Atlantis	Catch (per size and age class per species per spatial cell)		output	ES	Yes
Atlantis	Production rates		output	EF	Yes
Atlantis	Habitat extent		output	EF	Yes
Atlantis	Simple indices of biodiversity (Kempton Q, large fish index, size spectra slope, biomass rations between pelagic and demersal taxa)		output	biodiversity	NA
Atlantis	Environmental conditions - temperature		input	pressure	No
Atlantis	Environmental conditions - salinity		input	pressure	No
Atlantis	Environmental conditions - pCO2 in atmosphere		input	pressure	No
Atlantis	Environmental conditions - current flow		input	pressure	No

OSMOSE (Object-oriented Simulator of Marine ecOSystems Exploitation)	Biomass (per size and age class per species per spatial cell)		output	EF	Yes
OSMOSE (Object-oriented Simulator of Marine ecOSystems Exploitation)	Catch (per size and age class per species per spatial cell)		output	ES	Yes
OSMOSE (Object-oriented Simulator of Marine ecOSystems Exploitation)	Production rates		output	EF	Yes
OSMOSE (Object-oriented Simulator of Marine ecOSystems Exploitation)	Simple indices of biodiversity (Kempton Q, Large fish index)		output	biodiversity	NA
OSMOSE (Object-oriented Simulator of Marine ecOSystems Exploitation)	Primary production		input	EF	Yes
OSMOSE (Object-oriented Simulator of Marine ecOSystems Exploitation)	Environmental conditions (e.g. temperature)		input	pressure	No
SEAPODYM	Biomass (per age class per target species per spatial cell)		output	EF	Yes
SEAPODYM	Catch (per age class per target species per spatial cell)		output	ES	Yes
SEAPODYM	Primary production		input	EF	Yes

SEAPODYM	Environmental conditions (e.g. temperature)		input	pressure	No
APECOSM	Biomass (per age class per target species per spatial cell)		output	EF	Yes
APECOSM	Catch (per age class per target species per spatial cell)		output	ES	Yes
APECOSM	Primary production		input	EF	Yes
APECOSM	Environmental conditions (e.g. temperature)		input	pressure	No
LPJ-GUESS	Latitude, climate, soil texture, and CO2		input	pressure	No
LPJ-GUESS	Vegetation composition and cover	In the form of major species or plant functional types	output	biodiversity	NA
LPJ-GUESS	Biomass and soil organic matter carbon pools		output	EF	Yes
LPJ-GUESS	Leaf area index (LAI)		output	EF	Yes
LPJ-GUESS	Net primary production (NPP)		output	EF	Yes
LPJ-GUESS	Carbon emissions from wildfires		output	EF	No
LPJ-GUESS	Biogenic volatile organic compounds (BVOCs)		output	EF	Yes
LPJ-GUESS	Evapotranspiration		output	EF	Yes
LPJ-GUESS	Runoff		output	EF	Yes
LPJ-GUESS	Nitrogen pools and fluxes		output	EF	Yes
PREDICTS	Land use	Classes match IPCC's classification, including primary vegetation, secondary vegetation, plantation forest, cropland, urban, and pasture	input	pressure	No

PREDICTS	Land use intensity	The factors that determined this level depended on the land-use class. Classes include minimal, light, or intense use	input	pressure	No
PREDICTS	Land use history	number of years since the 30-arc-second grid cell containing each site became 30% covered by human land uses	input	pressure	No
PREDICTS	Human population density		input	pressure	Yes
PREDICTS	Proximity to roads		input	pressure	No
PREDICTS	Accessiblity from nearest large town		input	pressure	No
PREDICTS	[illegible] richness	[illegible] recorded at a given site in a standardised	output	biodiversity	NA
PREDICTS	total abundance	sum of the measures of abundance of all taxa at a site	output	biodiversity	NA
PREDICTS	rarefaction-based richness	1,000 random samples of n individuals from each site,where n is the smallest total number of individuals recorded at any site within its study, and calculating the mean species richness across samples	output	biodiversity	NA
PREDICTS	community-weighted mean plant height and animal mass	Plant height data were taken from the TRY database, animal body mass from the PanTHERIA database for mammals and BirdLife International's World Bird Database, and amphibians, reptiles, and arthropods from a wide range of published, unpublished, and grey-literature sources	output	biodiversity	NA
LPJ	Latitude, climate, soil texture, CO2		input	pressure	No
LPJ	Vegetation dynamics	populations of 10 plant functional types (8 woody and 2 herbaceous)	output	biodiversity	NA
LPJ	monthly net fluxes of CO2		output	pressure	Yes
LPJ	soil moisture levels		output	EF	No
LPJ	seasonal cycle of CO2 concentration		output	pressure	Yes
LPJ	global runoff		output	EF	No

LPJ	global carbon budgets and exchange	vegetation, soil, and litter pools	output	EF	Yes
LPJ	amplitude of seasonal cycle of CO2 at Mauna Loa		output	EF	No
LPJ	interannual exchange of CO2 between terrestrial and atmosphere		output	EF	No
LPJ	Net primary production (NPP)		output	EF	Yes
CABLE	Net primary production (NPP)		output	EF	Yes
CABLE	Vegetation dynamics	woody plant demography and dynamics	output	EF	Yes
CABLE	global terrestrial carbon balance		output	EF	Yes
CABLE	atmospheric CO2, climate variables		input	pressure	No
CABLE	land use change, land cover	gross land use transitions	input	pressure	No
CABLE	wood harvest		input	pressure	No
